# Non-Obese Diabetes and Its Associated Factors in an Underdeveloped Area of South China, Guangxi

**DOI:** 10.3390/ijerph13100976

**Published:** 2016-09-30

**Authors:** Zhenzhu Tang, Zhifeng Fang, Wei Huang, Zhanhua Liu, Yuzhu Chen, Zhongyou Li, Ting Zhu, Qichun Wang, Steve Simpson, Bruce V. Taylor, Rui Lin

**Affiliations:** 1 Guangxi Center for Disease Prevention and Control, 18 Jinzhou Road, Nanning 530028, China; tangzhzh@163.com (Z.T.); fangzhif80@126.com (Z.F.); hww_1989@sina.com (W.H.); hzliu326@sina.com (Z.L.); chenyz129@126.com (Y.C.); Lzy3030@126.com (Z.L.); ju20121225@163.com (T.Z.); wqchgxcdc@163.com (Q.W.); 2 Menzies Institute for Medical Research, University of Tasmania, Hobart 7001, Australia; steve.simpson@utas.edu.au (S.S.J.); bruce.taylor@utas.edu.au (B.V.T.); 3 Turning Point, Monash University, Fitzroy 3065, Australia

**Keywords:** diabetes, non-obese, prevalence, associated factors, underdeveloped area

## Abstract

Background: Little research has been conducted on the prevalence of diabetes mellitus in underdeveloped areas in China, especially stratified into obesity and non-obese diabetes. The aim of the present study was to investigate the prevalence and associated factors of non-obese diabetes in an underdeveloped area in South China, Guangxi. Methods: Data derived from the Chinese Health and Nutrition Survey 2010–2012 involved a sample of 3874 adults from Guangxi. Questionnaires and oral glucose-tolerance tests were conducted, and fasting and 2-h glucose levels and serum lipids were measured. Logistic regression analysis was performed to assess associated factors for non-obese diabetes. Results: 68.2% and 62.2% of instances of newly detected diabetes were those of non-obese diabetes based on BMI (NODB) and based on WC (NODW), respectively. The male sex, an age older than 50 years, lower education, hypertension, and hypertriglyceridemia were significantly associated with a higher risk of both NODB and NODW, while some associated factors for NODB were found different from those associated with NODW, and an interaction effect was found to increase the risk of NODW. Conclusions: Our study indicated that non-obese diabetes was highly prevalent in an underdeveloped area of South China. Non-obese diabetes should be considered for increased public attention in these areas.

## 1. Introduction

Diabetes mellitus (DM) has become a major public health problem in China: in the 2010 nationwide diabetes prevalence study, the prevalence of DM in China was found to be 11.6%, translating into 113.9 million adults with diabetes [1], of which above 90% would be type 2 diabetes [2]. DM has been considered a major risk factor for cardiovascular disease, the leading cause of death in China [3]. The aging of the Chinese population, increasing urbanization and concomitant nutritional changes, and decreasing levels of physical activity have led to an epidemic of obesity, which is a significant factor underlying the increasing diabetes burden in the Chinese population [4,5,6]. However, in underdeveloped areas such as Guangxi, a southern province in China, without this high prevalence of obesity [7], comparatively little research has been conducted on the prevalence of DM, especially stratified into obesity and non-obese diabetes. Interestingly, in Japan, over 60% of people with diabetes are not obese [8]. To address this gap in knowledge, we investigated and analyzed the prevalence and the associated factors for non-obese diabetes in an underdeveloped area in South China, Guangxi, from the China Nutrition and Health Survey conducted in 2010–2012 (CNHS 2010–2012).

## 2. Materials and Methods

### 2.1. Study Population

The data used in this study were derived from a part of the China Health and Nutrition Survey 2010–2012 (CHNS 2010–2012), a cross-sectional study conducted on the health and nutrition status of the Chinese population between 2010 and 2012. A multistage, stratified sampling method was used to select a representative sample of adults 18 years or older in the general population in Guangxi. The sampling process was stratified according to the degree of urbanization (one large city: the capital of Guangxi, Nanning City; one small city: Beihai City), and economic status (two developing counties: Binyang and Xingan; one underdeveloped county: Lingyu) in Guangxi. The first two stages of sampling were selected by economic status, and cities and counties were not random. In the next two stages (the stage in which districts were selected from cities and rural townships from counties and the stage in which street districts were selected from city districts and rural villages from townships), the sampling was random. In total, 12 urban street districts and 18 rural villages were selected. Survey protocols and the procedures were approved by the Ethics Committee of the National Institute of Nutrition and Health, Chinese Center for Disease Control and Prevention (2013-018). Written informed consent was obtained from each participant before data collection. Additional details regarding the CHNS 2010–2012 are provided elsewhere [9,10,11].

A total of 3874 people participated in the study, with data on fasting or 2-h plasma glucose levels. Considering that those with a current diabetes diagnosis might have changed their diet or lifestyle after diagnosis, we excluded 131 such prediagnosed cases, leaving 3743 persons for analysis of newly detected diabetes.

### 2.2. Data Collection

A standard questionnaire was administered by trained staff to obtain information on demographic characteristics, personal and family medical history, socioeconomic status, educational level, occupation, and lifestyle (e.g., smoking, drinking, and physical activity). The interview included questions related to the diagnosis and treatment of diabetes, hypertension, dyslipidemia, and cardiovascular events. Cigarette smoking was classified as current smoking and former smoking vs. never. Information on the amount per week and type of alcohol consumed during the previous year was obtained. Physical activity level (PAL) was calculated using the formula published by the Food and Nutrition Board of the Institute of Medicine, USA [12], as following by calculated using individual metabolic equivalent (MET) [13].

For male: ΔPAL = [(A·METs − 1) × 1.34 × (B min)/1440 min,(1)

For female: ΔPAL = [(A·METs − 1) × 1.42 × (B min)/1440 min, and(2)

PAL = 1.0 + ΔPAL,(3)

where A refers to the intensity of one activity, and B refers to the time of the activity. Sufficient physical activity was defined as PAL ≥ 1.7, and insufficient physical activity as PAL < 1.7.

The economic development of cities or counties was defined on the basis of the per capita GDP in 2009. Blood pressure, body weight, height, and waist circumference (WC) were measured using standard methods, as described elsewhere [2].

### 2.3. Oral Glucose-Tolerance Test

Blood samples were collected by the study nurses after an overnight fast of at least 8 h, and a venous blood specimen was collected for the measurement of plasma glucose. Participants without a history of diabetes were then given a standard 75-g glucose tolerance test with plasma glucose measured again at 2 h post-oral glucose load. Plasma glucose was measured using the hexokinase enzymatic method, and triglycerides (TG), high-density lipoprotein cholesterol (HDL-cholesterol), and low-density lipoprotein cholesterol (LDL-cholesterol) were assessed enzymatically using commercially available reagents (Shanghai Kehua Bio-engineering Co., Ltd., Shanghai, China) on an automatic analyzer (Hitachi 7080; Hitachi, Tokyo, Japan) at the clinical biochemical laboratory in the Guangxi Center for Disease Prevention and Control, China.

### 2.4. Definition of Outcomes

The 1999 World Health Organization diagnostic criteria were used to diagnose diabetes [14]. Briefly, the criteria define diabetes as a fasting glucose level ≥7.0 mmol/L, a 2-h glucose level in the glucose-tolerance test ≥11.1 mmol/L, or both. Previously diagnosed diabetes was identified by a positive response from the participant to the question, “Has a doctor ever told you that you have diabetes?”, their taking oral glucose-lowering agents within the previous two weeks, or both. The total number of diabetes patients included prediagnosed and newly detected cases.

Overweight was defined according to standard WHO criteria as 25 kg/m^2^ ≤ BMI < 30 kg/m^2^, and obesity was defined as BMI ≥ 30 kg/m^2^. Abdominal obesity was defined as waist circumference (WC) ≥ 90 cm for males and WC ≥ 80 cm for females based on revised Asian criteria [15]. According to Chinese diagnostic criteria of dyslipidemia [16], hypercholesteremia was defined as total cholesterol (TC) ≥6.22 mmol/L (240 mg/dL), hypertriglyceridemia as triglycerides (TG) ≥2.26 mmol/L (200 mg/dL), low HDL-cholesterol as HDL-cholesterol <1.04 mmol/L (40 mg/dL), and high LDL-cholesterol as LDL-cholesterol ≥4.14 mmol/L (160 mg/dL). Anemia was defined as hemoglobin <130 g/L for males and hemoglobin <120 g/L for non-pregnant females aged over 15 years.

In this context, non-obese diabetes refers to those newly detected DM patients with a normal WC (male < 90 cm, female < 80 cm, NODW) or without an overweight/obese BMI (BMI < 25 kg/m^2^, NODB).

### 2.5. Statistical Analysis

Demographic and metabolic characteristics of study participants were described in percentage, and all the variables were stratified as categorical ones including those continuous variables based on the definition of outcome. Prevalence estimated for newly detected diabetes mellitus (NEDDM) as calculated for overall population and non-obese diabetes (NODW and NODB) were calculated for those populations with normal WC and those without overweight or obesity, respectively.

A stepwise logistic regression was used to examine the association of sociodemographic, family medical history, lifestyle, and metabolic factors with the odds of diabetes. With the use of backward elimination, only covariates that were significant (*p* < 0.1) were retained in the final model. To examine whether there was an interaction between covariates’ association with diabetes, a product term was included in the model, the significance of which delineated the significance of interaction.

All statistical analyses were performed using STATA 12.0 (Stata Corp LP, College Station, TX, USA).

## 3. Results

### 3.1. Prevalence of Non-Obese Diabetes

Among the diabetes patients, 64.0% (233/364) of individuals had newly detected diabetes mellitus (NEDDM), of which 68.2% (159/233) were non-obese diabetes based on BMI (NODB, BMI < 25 kg/m^2^), and 62.2% (145/233) were non-obese diabetes based on WC (NODW, male < 90 cm, female < 80 cm).

**Figure 1:**
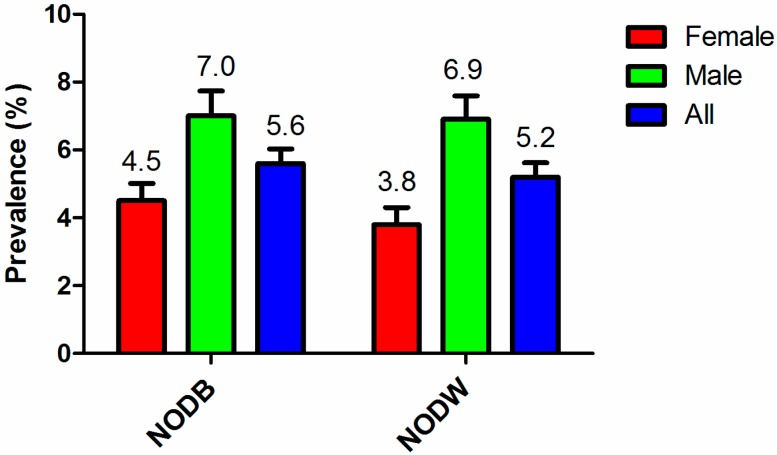
The prevalence of non-obese diabetes based on BMI (NODB) and waist circumference (WC) (NODW) among males and females. A significantly higher prevalence was observed in males than in females, both for NODB and NODW (*p* < 0.05 with chi-square test).

**Figure 2:**
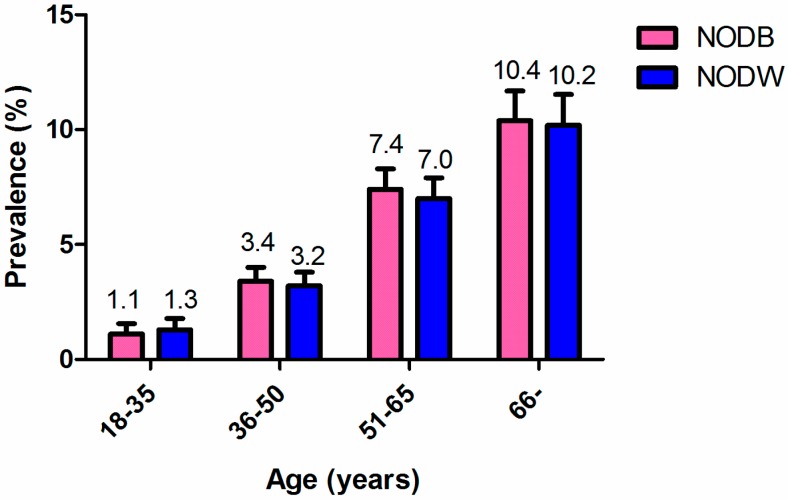
The prevalence of non-obese diabetes based on BMI (NODB) and WC (NODW) according to age groups. Both the prevalence of NODB and NODW increased with age.

The prevalence of diabetes mellitus and NEDDM were 9.4% (364/3874) and 6.2% (233/3743), respectively. Among the population with normal WC or without overweight or obesity, the prevalence of NODW and of NODB were 5.2% (145/2763) and 5.6% (159/2852), respectively. The prevalence of diabetes was significantly higher in males than in females, both for NODB and for NODW (Figure 1), and both the prevalence of NODB and NODW increased with age (Figure 2).

### 3.2. Characteristics of Non-Obese Diabetes Mellitus

Participants detected as NODB or NODW were more likely to be Han people, married/divorced, and living in rural areas, and a lower proportion had completed junior or high school or above, had a lower income (<20,000/year), and reported less physical activity. Notably, about 47% of NODB and 45% of NODW patients had hypertension; about 44% of NODB and 50% of NODW subjects reported routinely drinking alcohol; 29% of NODB and 35% of NODW patients were current smokers; and 30% of NODB and 25% of NODW patients had hypertriglyceridemia. Supporting that both were correctly allocated to non-obese diabetes, for NODB patients, 19.5% had abdominal obesity, and 11.7% of NODW patients were overweight or obesity based on BMI (Table 1).

### 3.3. Factors Associated with NODB and NODW

As shown in Table 2, the male sex, an older age, being married, divorced, or widowed, hypertension, hypertriglyceridemia, and low HDL-cholesterol were significantly associated with a higher risk of NODB, while a higher education was significantly associated with a lower risk of NODB in the univariable analysis (*p* < 0.05); besides Zhuang ethnicity, higher income and abdominal obesity was marginally associated with NODB status (0.05 < *p* < 0.1). After adjustment for covariates, the male sex, an age older than 50 years, an education level lower than junior high school, hypertension, hypertriglyceridemia, and abdominal obesity remained positively associated with NODB status (*p* < 0.05), while Zhuang ethnicity persisted as marginally negatively associated with NODB (OR: 0.63; 95% CI: 0.38, 1.02; *p* = 0.058), and the associations between NODB and being married, divorced, or widowed and low HDL-cholesterol disappeared. As for NODW, the male sex, an older age, being married, divorced, or widowed, smoking, drinking alcohol, hypertension, hypertriglyceridemia, and low HDL-cholesterol were significantly associated with a higher risk of NODW, while higher education, higher income, and higher BMI were significantly associated with a lower risk of NODW in the univariable analysis (*p* < 0.05). After adjustment, an age older than 50 years, hypertension, and hypertriglyceridemia persisted in their associations with a higher risk of NODW (*p* < 0.05), while other associations either became attenuated (e.g., the male sex, an education level with junior high school, high annual income, and low HDL) or were abrogated.

Interestingly, the factors associated with NODB were found to be different from those associated with NODW status. For example, Zhuang ethnicity was negatively associated with NODB prevalence compared with Han people, while no association was found with NODW. Meanwhile, higher income and low HDL was associated with NODW status, but no similar association was seen for NODB. Furthermore, the male sex was significantly associated with a higher risk of NODB, while only a marginal association was found with NODW.

### 3.4. Interaction Effect on NODW

We found that, while smoking was not independently associated with the risk of NODW, it significantly modulated the relationship between TG and NODW, such that no significant association was observed between TG and NODW when the NODW patients were non-smokers (OR 1.43, 95% CI: 0.74, 2.77; *p* = 0.29), while a positive association was observed between TG and NODW when the NODW patients were smokers (OR 3.25, 95% CI: 1.60, 6.59; *p* = 0.001), with a *p*_interaction_ = 0.026. However, no modulating effect was found on the relationship between TG and NODB when stratified by smoking status, such that a significant positive association was observed when the NODB patients were both non-smokers (OR 2.25, 95% CI: 1.32, 3.83; *p* = 0.003) and smokers (OR 4.10, 95% CI: 1.93, 8.70; *p* < 0.001), with no interaction effect found (*p*_interaction_ = 0.27) (Table 3).

## 4. Discussion

In Guangxi, from the China Health and Nutrition Survey 2010–2012, we have shown that 68.2% and 62.2% of instances of newly detected diabetes mellitus were those of non-obese diabetes based on BMI (NODB) and WC (NODW), respectively. The male sex, an age older than 50 years, lower education, hypertension, and hypertriglyceridemia were significantly associated with a higher risk of both NODB and NODW, while Zhuang ethnicity was marginally associated with a lower risk of NODB compared with Han people, the majority ethnicity in China, and a higher income showed a marginally protective association with NODW. Moreover, for those factors associated with NODW, we demonstrated that the relationship between TG and NODW was modulated when the patients were smokers.

We found that more than 60% of instances of newly detected diabetes mellitus were those of non-obese diabetes, whether they were based on BMI or based on WC, which is consistent with the results from Japan, where over 60% of the diabetic subjects were not obese [8]. Previously, studies on obesity and diabetes in Asian populations showed the prevalence of overweight and obesity in Asians was lower than that in Europeans and Americans [17,18]. In our study, it was not very common for participants to be obese (2.6%) or overweight (20.6%) based on BMI, which was lower than that of Chinese or American general populations (mean BMI 22.8 in our study vs. 23.7 in the Chinese general population [1] and 28.7 in the U.S. population [18]). Given the low urbanization in underdeveloped areas such as Guangxi, the lifestyle (e.g., dietary, physical activity) may not have equilibrated to those seen in more urban areas of China or those seen in Western nations like the USA. This finding suggests that anti-diabetes interventions should be considered at all levels of BMI in Guangxi. This is particularly the case for non-obese diabetes, which is a presentation not typically monitored by medical surveillance but which should be considered for increased public attention.

In Asia, research has shown that people with a “metabolically obese” phenotype (e.g., normal body weight with increased abdominal adiposity) was common [19]. In this study, abdominal obesity was shown to be independently associated with a higher risk of NODB. This is concerning since, even among persons with normal BMI, central abdominal obesity—a parameter not routinely measured—may yet place them at risk for non-obese diabetes. Indeed, abdominal obesity has been demonstrated to be the best predictor of type 2 diabetes compared to BMI, waist/hip ratio, and other anthropometric measurements [20].

However, it is interesting to find that non-obese diabetic individuals had low proportions of hypertension (7.3% and 6.5%), hypertriglyceridemia (8.2% and 6.3%), and low HDL-cholesterol (6.9% and 5.8%) for NODB and NODW, respectively in the study. These findings suggest that apart from cardio-metabolic factors, other factors may contribute to the prevalence of non-obese diabetes in Guangxi, such as genetic predispositions. Zhuang ethnicity was found to be near-significantly associated with a lower risk of NODB in this study. Given that Guangxi is the largest Zhuang autonomous region in China, and different genetics among different race and ethnic groups contributing to obesity and type 2 diabetes have been documented [21,22,23], a different genetic background may explain some of the differences seen in this study from others in China/Asia and abroad. In Japan, non-obese type 2 diabetes patients have been demonstrated as having a stronger genetic predisposition to type 2 diabetes than obese type 2 diabetes [24]. Whether the genetic predisposition has a stronger effect on non-obese type 2 diabetes in Guangxi requires further study.

Our study has several limitations. First, we did not distinguish between type 1 and type 2 diabetes in this study. Nevertheless, type 2 diabetes is the predominant form of diabetes in adults [2], and we can extrapolate onto our sample that the majority of cases are likely type 2 diabetes. Second, missing data for some participants would potentially introduce information bias, although the large numbers recruited should reduce this risk. Thirdly, dietary intake was not collected, so we were unable to analyze dietary contributions to diabetes prevalence. The strength of the study is the multistage, stratified sampling method to select the sample, leading to good representative participants for the study in this underdeveloped area of South China.

At present, rural China is facing an increase in the incidence of cardiovascular disease [1]. Type 2 diabetes and hypertension are key risk factors for cardiovascular disease [25,26]. Here, we found that hypertension and hypertriglyceridemia, cardio-metabolic markers, were positively associated with both NODB and NODW prevalence, and low HDL-cholesterol was marginally associated with a higher risk of NODW. Moreover, a modulating effect was found for NODW, with smoking interacting with triglyceride levels to increase the risk of NODW. Our findings suggest that an integrated management is required to address rural China’s increasing diabetes rates and to consequently reduce the cardiovascular disease burden.

## 5. Conclusions

In summary, our results showed that non-obese diabetes was highly prevalent in the general adult population in the underdeveloped Guangxi province of South China. Given the large population at risk and that the majority of cases of diabetes being undiagnosed and non-obese, this would suggest that the prevention and screening strategies for diabetes in Guangxi need to cover the general population at all levels of BMI. Similarly, the known risk factors for type 2 diabetes in Western and urban areas of China to some extent do not have as significant role in the risk of non-obese type 2 diabetes in rural South China; therefore, standard prevention strategies may not have the same effects on prevention in this population.

## Figures and Tables

**Table 1:** Characteristics of newly diagnosed and non-obese diabetes mellitus in Guangxi.

Characteristics	No. of Participants (%) (*n* = 3743)	No. of NODB ^a^ (%) (*n* = 159)	No. of NODW ^b^ (%) (*n* = 145)
Sex			
Female	2160 (57.7)	75 (47.2)	54 (37.2)
Male	1583 (42.3)	84 (52.8)	91 (62.8)
Age (years)			
18–35	633 (16.9)	6 (3.8)	7 (4.8)
36–50	1201 (32.1)	30 (18.9)	29 (20.0)
51–65	1167 (31.2)	64 (40.3)	56 (38.6)
66–	742 (19.8)	59 (37.1)	53 (36.6)
Ethnicity			
Han	2827 (75.5)	130 (81.8)	113 (77.9)
Zhuang	778 (20.8)	25 (15.7)	28 (19.3)
Others	138 (3.7)	4 (2.5)	4 (2.8)
Education			
Primary school and below	1595 (42.6)	96 (60.4)	83 (57.2)
Junior high school	1410 (37.7)	36 (22.6)	36 (24.8)
Senior high school and above	738 (19.7)	27 (17.0)	26 (17.9)
Per-capita annual income (Yuan) *			
＜5000	1000 (27.7)	47 (30.1)	50 (35.2)
5000–9999	1064 (29.5)	43 (27.6)	32 (22.5)
10,000–19,999	1041 (28.9)	52 (33.3)	47 (33.1)
≥20,000	503 (13.9)	14 (9.0)	13 (9.2)
Marriage status			
Unmarried	208 (5.6)	2 (1.3)	2 (1.4)
Married	3174 (84.8)	137 (86.2)	125 (86.2)
Divorced/Widowed	361 (9.6)	20 (12.6)	18 (12.4)
Residence			
Urban	1497 (40.0)	68 (42.8)	63 (43.4)
Rural	2246 (60.0)	91 (57.2)	82 (56.6)
Physical activity level *			
Sufficient	1126 (43.2)	51 (45.9)	43 (42.6)
Insufficient	1479 (56.8)	60 (54.1)	58 (57.4)
Smoking *			
No	2794 (75.2)	113 (71.1)	95 (65.5)
Yes	920 (24.8)	46 (28.9)	50 (34.5)
Alcohol *			
No	2262 (60.8)	89 (56.0)	73 (50.3)
Yes	1458 (39.2)	70 (44.0)	72 (49.7)
Hypertension *			
No	2720 (73.0)	85 (53.5)	80 (55.2)
Yes	1007 (27.0)	74 (46.5)	65 (44.8)
Hypertriglyceridemia *			
No	3107 (84.4)	110 (70.1)	107 (74.8)
Yes	575 (15.6)	47 (29.9)	36 (25.2)
Hypercholesterolemia *			
No	2876 (78.3)	123 (78.3)	118 (82.5)
Yes	799 (21.7)	34 (21.7)	25 (17.5)
Low HDL ^c,^*			
No	3118 (84.7)	118 (75.2)	110 (76.9)
Yes	565 (15.3)	39 (24.8)	33 (23.1)
High LDL ^d,^*			
No	3131 (85.1)	137 (87.3)	127 (88.8)
Yes	550 (14.9)	20 (12.7)	16 (11.2)
Anemia *			
No	3448 (92.3)	143 (89.9)	131 (90.3)
Yes	288 (7.7)	16 (10.1)	14 (9.7)
BMI (kg/m^2^) ^e,^*			
<25.0	2852 (76.7)	159 (100.0)	128 (88.3)
≥25.0	866 (23.3)	-	17 (11.7)
Abdominal obesity *			
No	2763 (74.4)	128 (80.5)	145 (100.0)
Yes	950 (25.6)	31 (19.5)	-
Family history of hypertension *			
No	2052 (78.3)	79 (74.5)	70 (75.3)
Yes	570 (21.7)	27 (25.5)	23 (24.7)
Family history of coronary heart disease *			
No	2347 (96.1)	90 (94.7)	80 (95.2)
Yes	96 (3.9)	5 (5.3)	4 (4.8)
Family history of cerebral apoplexy *			
No	2342 (95.8)	91 (94.8)	81 (95.3)
Yes	103 (4.2)	5 (5.2)	4 (4.7)
Family history of diabetes mellitus *			
No	2332 (95.2)	93 (97.9)	82 (97.6)
Yes	118 (4.8)	2 (2.1)	2 (2.4)

^a^ NODB = non-obese diabetes (BMI ≤ 25 kg/m^2^); ^b^ NODW = non-obese diabetes (waist circumference (WC) < 90 cm for males and WC < 80 for females); ^c^ HDL = high density lipoprotein; ^d^ LDL = low density lipoprotein; ^e^ BMI = body mass index. * *n* = 3608, *n* = 2605, *n* = 3714, *n* = 3720, *n* = 3727, *n* = 3682, *n* = 3675, *n* = 3683, *n* = 3681, *n* = 3736, *n* = 3718, *n* = 3713, *n* = 2622, *n* = 2443, *n* = 2445, and *n* = 2450 for per-capita annual income, physical activity level, smoking, alcohol, hypertension, hypertriglyceridemia, hypercholesterolemia, low HDL, high LDL, anemia, BMI, abdominal obesity, family history of hypertension, coronary heart disease, cerebral apoplexy and diabetes mellitus, respectively, because of missing values.

**Table 2:** Associated factors for non-obese diabetes in Guangxi.

Factor	Univariable Analysis				Full Model			
	NODB		NODW		NODB		NODW	
	OR (95% CI)	*p* Value	OR (95% CI)	*p* Value	OR (95% CI)	*p* Value	OR (95% CI)	*p* Value
Sex								
Female	1.00 (ref)		1.00 (ref)		1.00 (ref)		1.00 (ref)	
Male	1.56 (1.13, 2.14)	0.006	2.38 (1.69, 3.35)	<0.001	1.49 (1.02, 2.17)	0.039	1.58 (0.98, 2.55)	0.059
Age (years)								
18–35	1.00 (ref)		1.00 (ref)		1.00 (ref)		1.00 (ref)	
36–50	2.68 (1.11, 6.47)	0.029	2.21 (0.96, 5.08)	0.061	2.00 (0.78, 5.16)	0.149	1.95 (0.81, 4.73)	0.139
51–65	6.06 (2.61, 14.08)	<0.001	4.51 (2.04, 9.95)	<0.001	3.41 (1.35, 8.62)	0.010	3.68 (1.55, 8.75)	0.003
66–	9.03 (3.87, 21.05)	<0.001	6.90 (3.11, 15.29)	<0.001	4.65 (1.77, 12.18)	0.002	5.30 (2.14, 13.11)	<0.001
Ethnicity								
Han	1.00 (ref)		1.00 (ref)		1.00 (ref)		-	
Zhuang	0.69 (0.45, 1.06)	0.093	0.90 (0.59, 1.37)	0.610	0.63 (0.38, 1.02)	0.058	-	-
Others	0.62 (0.23, 1.70)	0.352	0.72 (0.26, 1.97)	0.518	0.54 (0.18, 1.56)	0.252	-	-
Education								
Primary school and below	1.00 (ref)		1.00 (ref)		1.00 (ref)		1.00 (ref)	
Junior high school	0.41 (0.28, 0.60)	<0.001	0.48 (0.32, 0.71)	<0.001	0.54 (0.35, 0.85)	0.007	0.64 (0.41, 1.00)	0.050
Senior high school and above	0.59 (0.38, 0.92)	0.019	0.66 (0.42, 1.04)	0.074	0.91 (0.55, 1.49)	0.700	1.03 (0.62, 1.71)	0.941
Per-capita annual income (Yuan)								
<5000	1.00 (ref)		1.00 (ref)		1.00 (ref)		1.00 (ref)	
5000–9999	0.85 (0.56, 1.30)	0.464	0.59 (0.37, 0.93)	0.022	0.97 (0.62, 1.52)	0.900	0.68 (0.43, 1.10)	0.114
10,000–19,999	1.07 (0.71, 1.60)	0.756	0.90 (0.60, 1.35)	0.607	1.30 (0.84, 2.01)	0.235	1.09 (0.71, 1.69)	0.688
≥20,000	0.58 (0.32, 1.06)	0.079	0.50 (0.27, 0.94)	0.030	0.67 (0.35, 1.30)	0.237	0.54 (0.28, 1.05)	0.068
Marital status								
Unmarried	1.00 (ref)		1.00 (ref)		1.00 (ref)		1.00 (ref)	
Married	4.65 (1.14, 18.90)	0.032	4.23 (1.04, 17.20)	0.044	2.03 (0.44, 9.31)	0.360	1.85 (0.41, 8.36)	0.425
Divorced/Widowed	6.04 (1.40, 26.11)	0.016	5.41 (1.24, 23.53)	0.025	1.66 (0.33, 8.35)	0.541	1.48 (0.29, 7.44)	0.637
Smoking								
No	1.00 (ref)		1.00 (ref)		-		1.00 (ref)	
Yes	1.25 (0.88, 1.77)	0.215	1.63 (1.15, 2.32)	0.006	-	-	0.85 (0.54, 1.34)	0.493
Alcohol								
No	1.00 (ref)		1.00 (ref)		-		1.00 (ref)	
Yes	1.23 (0.89, 1.70)	0.203	1.56 (1.12, 2.17)	0.009	-	-	1.04 (0.70, 1.56)	0.838
Hypertension								
No	1.00 (ref)		1.00 (ref)		1.00 (ref)		1.00 (ref)	
Yes	2.46 (1.78, 3.39)	<0.001	2.28 (1.63, 3.19)	<0.001	1.83 (1.26, 2.65)	0.002	1.58 (1.07, 2.32)	0.022
Hypertriglyceridemia								
No	1.00 (ref)		1.00 (ref)		1.00 (ref)		1.00 (ref)	
Yes	2.43 (1.70, 3.45)	<0.001	1.87 (1.27, 2.76)	0.002	2.67 (1.75, 4.07)	<0.001	2.08 (1.31, 3.30)	0.002
Low HDL								
No	1.00 (ref)		1.00 (ref)		1.00 (ref)		1.00 (ref)	
Yes	1.89 (1.30, 2.74)	0.001	1.70 (1.14, 2.53)	0.009	1.42 (0.91, 2.21)	0.126	1.52 (0.95, 2.43)	0.080
BMI (kg/m^2^)								
<25	-		1.00 (ref)		-		1.00 (ref)	
≥25	-	-	0.43 (0.26, 0.71)	0.001	-	-	1.20 (0.70, 2.12)	0.531
Abdominal obesity								
No	1.00 (ref)		-		1.00 (ref)		-	
Yes	0.69 (0.47, 1.04)	0.074	-	-	1.63 (1.01, 2.63)	0.044	-	-

**Table 3:** The association between triglycerides (TG) and NODW, NODB stratified by smoking status that modified the TG–NODW and TG–NODB association.

	NODW			NODB		
Smoking Status	No. of Patient	OR (95% CI)	*p* Value	No. of Patient	OR (95% CI)	*p* Value
Non-smoker	95	1.43 (0.74, 2.77)	0.29	113	2.25 (1.32, 3.82)	0.003
* Smoker	50	3.25 (1.60, 6.59)	0.001	46	4.10 (1.93, 8.70)	<0.001
		2.69 (1.13, 6.43)	*p*_interaction_ = 0.026		1.59 (0.70, 3.61)	*p*_interaction_ = 0.27

* Smoker includes former smoker and current smoker, in which the number of former smokers (*n* = 10 with NODW) and (*n* = 9 with NODB) is combined with the number of current smoker because of the small proportion. *p*_interaction_ was assessed in the full model.
